# A computer vision-based approach for automatically extracting data from bar chart raster images to facilitate meta-analysis of biomedical literature

**DOI:** 10.1371/journal.pone.0347081

**Published:** 2026-07-31

**Authors:** Alexander Cardaras, Seungjun Kim, Ye Yuan, Itamar Livnat, Ryan T. Yanagihara, Rosita Saul, Gabrielle Montes De Oca, Kai Zheng, Andrew W. Browne

**Affiliations:** 1 Department of Computer Science, Donald Bren School of Information and Computer Sciences, University of California, Irvine, California, United States of America; 2 Department of Informatics, Donald Bren School of Information and Computer Sciences, University of California, Irvine, California, United States of America; 3 Department of Ophthalmology, Los Angeles General Medical Center, Los Angeles, California, United States of America; 4 Department of Ophthalmology, University of Washington, Seattle, Washington, United States of America; 5 Department of Biological Sciences, Schmid College of Science and Technology, Chapman University, Orange, California, United States of America; 6 Brunson Center for Translational Vision Research, University of California, Irvine, California, United States of America; 7 Department of Biomedical Engineering, University of California, Irvine, California, United States of America; 8 Department of Ophthalmology, University of California, Irvine, California, United States of America; Icahn School of Medicine at Mount Sinai, UNITED STATES OF AMERICA

## Abstract

Although bar charts are widely used in scientific publications, their rasterized format within Portable Document Format (PDF) files complicates automated data extraction, hindering large-scale evidence synthesis and meta-analysis. To address this, we developed and evaluated an automated pipeline for extracting quantitative data from bar charts embedded in the biomedical literature. The four-stage pipeline comprises (1) image extraction and panel segmentation, (2) optical character recognition (OCR)-based text detection, (3) image disassembly to identify chart components, and (4) data reconstruction using numeric parsing and axis-based interpolation. The system combines edge detection, morphological operations, and convolutional neural network (CNN)-based figure classification using a transfer-learned Inception v3 model. Performance was validated on randomized controlled trials in age-related macular degeneration, with manually annotated values from a semi-automated labeling tool as the reference standard, and agreement was assessed using Bland–Altman analysis. Across 28 bar charts from ten publications, the pipeline correctly recognized 92.9% (95% confidence interval [CI], 77.4–98.0) of figure types, 96.0% (95% CI, 94.2–97.3) of text blocks, and 79.1% (95% CI, 74.7–83.0) of bars. For numerical reconstruction, 81.2% (95% CI, 76.3–85.2) of bar values fell within ±5% of the reference standard, 63.0% (95% CI, 57.3–68.3) within ±2%, and 48.6% (95% CI, 43.0–54.3) within ±1%. Bland–Altman analysis showed a small negative bias of −0.18 (95% CI, −0.34 to −0.02), with 94.9% of differences within the limits of agreement. Most outliers arose from OCR digit misclassification or ambiguous bar boundaries. This proof-of-concept study demonstrates the feasibility of automated data extraction from bar charts using a hybrid approach that combines image-processing heuristics with CNN-based classification. Although currently limited to bar charts and a single clinical domain, the pipeline represents a step toward scalable, end-to-end systems for automated evidence extraction to support meta-analyses across the biomedical literature.

## Introduction

The volume of biomedical literature has expanded at an unprecedented rate in recent decades. MEDLINE added nearly one million new citations in 2020 alone, and PubMed now indexes more than 38 million citations and abstracts, making scalable evidence synthesis increasingly important [1]. This expanding body of literature represents a vast repository of biomedical knowledge spanning molecular biology, clinical medicine, behavioral science, and population health.

Individual studies frequently lack sufficient statistical power to yield conclusive, generalizable evidence capable of informing evidence-based clinical practice [2]. As a result, systematic reviews and meta-analyses have become essential for synthesizing evidence, allowing researchers to aggregate findings across studies and derive more robust conclusions. However, the data extraction required for these syntheses remains a major methodological bottleneck. The absence of standardized, machine-readable reporting formats across most publications requires labor-intensive manual extraction of numerical results, effect sizes, and study characteristics from full-text articles [3]. This challenge is compounded by the predominant use of the Portable Document Format (PDF), a presentation-oriented format that lacks semantic structure and impedes automated data extraction [4]. The resulting manual curation process is time-consuming, resource-intensive, prone to human error, and difficult to scale as the published literature continues to grow.

Although computational tools have been developed to support evidence synthesis, most have focused on extracting information from article text or tables rather than figures [5,6]. This leaves an important gap, because health-science publications often report crucial quantitative results only in raster images, such as bar charts, histograms, line graphs, and scatter plots [7]. When such figures are embedded in PDF articles, their underlying numerical values are not directly accessible to automated review systems. Methods that combine computer-vision techniques, OCR, and deep-learning models such as CNNs are therefore needed to convert figure-embedded results into structured, machine-readable data.

Automated data extraction from chart images is not new. Huang and Tan proposed a system for understanding imaged infographics of bar and pie charts, achieving 93.75% graphics-recognition accuracy and 88.22% text-block classification accuracy on bar-chart images [8]. He et al. combined hand-coded rules with a CNN for bar-chart mining in PubMed Central but correctly recognized only 39.81% of bar panels [9]. Liu et al. used a Faster R-CNN object-detection model to identify chart components such as text and axes and then applied relational-network components to infer coordinate–value relationships, achieving a 64.75% recognition rate [7]. More recent work in the machine-learning community has framed chart understanding as an end-to-end learning problem: ChartOCR extracts underlying data from bar, line, and pie charts using a deep key point-detection and rule-based hybrid; ChartQA benchmarks visual and logical reasoning over charts; and DePlot translates plots into structured tables to support downstream reasoning by large language models [10–12]. In parallel, semi-automated tools such as BarChartAnalyzer integrate chart-type classification, object and text detection, and tensor-voting–based data extraction with optional summarization [13,14]. Despite this progress, comparatively little work has targeted fully automated extraction of quantitative data from bar charts in the biomedical literature, where high precision is required to prevent the propagation of errors into downstream meta-analytic calculations. Comprehensive data extraction from bar charts requires accurate identification of several interconnected components, including individual bars, coordinate axes, axis scales and labels, bar-specific annotations, embedded text, and the quantitative values implied by bar dimensions relative to axis scales. The development of end-to-end systems that transform graphical representations into structured, machine-readable formats with clinically acceptable accuracy therefore remains an unmet need in biomedical informatics.

To address these gaps, we developed and evaluated an automated computer-vision pipeline for extracting quantitative data from bar charts embedded in the biomedical literature. We focused on bar charts because they are among the most common formats for presenting numerical study outcomes, yet their rasterized format within PDF documents typically prevents direct data extraction. Unlike general-purpose chart-understanding systems, our work is motivated by a specific evidence-synthesis use case: converting quantitative information locked in biomedical figure images into structured data that can support systematic reviews and meta-analyses. To demonstrate the pipeline’s utility and assess its performance, we conducted a proof-of-concept meta-analysis of visual-acuity outcomes in randomized controlled trials (RCTs) of age-related macular degeneration (AMD) treatments. AMD, a progressive retinal degenerative disease and a leading cause of blindness among elderly populations in developed countries [15–19], is commonly treated with laser-based therapies or anti–vascular endothelial growth factor (anti-VEGF) agents, including bevacizumab, ranibizumab, aflibercept, and brolucizumab [20]. We selected AMD as the test case because of its well-defined outcome measures (visual acuity and central foveal thickness), the substantial volume of published RCTs, and the clinical importance of synthesizing evidence across trials. Importantly, the pipeline architecture is disease-agnostic and readily adaptable to evidence synthesis across diverse medical conditions and clinical domains.

## Materials and methods

### Architecture of the data extraction pipeline

**Fig 1:**
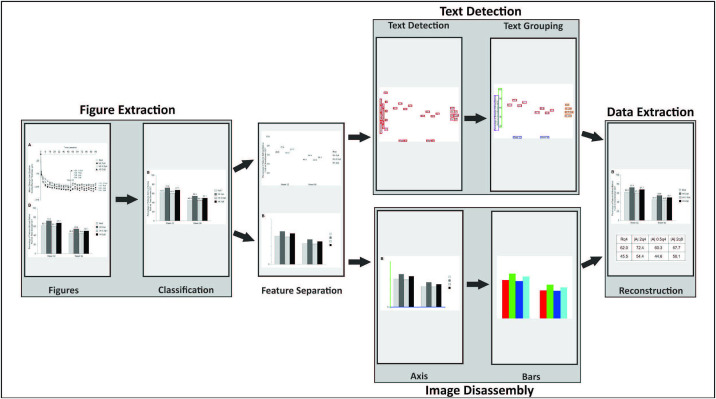
Automated data extraction pipeline.

Our data extraction pipeline consists of four steps: (1) raster image extraction and subgraph separation, (2) text detection, (3) image disassembly, and (4) data extraction (Fig 1). During figure extraction, the algorithm scrubs figures and images from a document and categorizes them by figure type (bar graph versus line graph versus other). Text recognition then preprocesses each image and performs optical character recognition (OCR). Structural features such as bars, axes, and legends are identified during image disassembly. Finally, the chart is reconstructed by pairing recognized text with the feature objects identified during data extraction to produce tabulated data that can be readily analyzed in meta-analyses.

Fig 1 presents a high-level overview of the pipeline. The detailed configuration of each module is described in the subsections below. In Stage 1, MuPDF extracts rasterized images, multi-panel figures are segmented into constituent panels, and each panel is classified with an Inception v3 CNN adapted by transfer learning. The classifier takes 299 × 299-pixel RGB panels as input and retains the convolutional base pretrained on ImageNet, while the final fully connected and softmax layers are replaced and retrained to output figure-type probabilities (bar chart, line graph, scatter plot, or other); only panels classified as bar charts proceed downstream. Stage 2 performs text detection through a preprocessing chain—Otsu binarization, contour-based removal of large graphical elements, text-mask construction, and Waifu2x super-resolution—followed by Tesseract OCR. Stage 3 recovers chart geometry using Canny edge detection and a probabilistic Hough transform to localize the axes, morphological opening to suppress gridlines, and a contour-pairing procedure to reconstruct individual bars, with template and color matching to group multi-series bars. Stage 4 integrates the recognized text with the reconstructed geometry, classifies text by orientation and position into axis labels and tick values, and computes each bar value from a directly annotated label when present or, otherwise, from a linear pixel-to-value mapping calibrated on the y-axis tick spacing. The pipeline is therefore modular and predominantly rule-based, with a single learned component—the figure-type classifier—acting as a panel-level gate.

### Raster image extraction and subgraph separation

The pipeline begins by identifying all figures embedded in the full text of each article, including figures available only within PDF documents. We first extract rasterized images using MuPDF, a lightweight open-source framework capable of batch-processing large document collections and exporting images into formats such as Joint Photographic Experts Group (JPEG) or Portable Network Graphics (PNG) [21]. Each image then undergoes constituent-panel segmentation to isolate individual subfigures—a critical step, because many scientific figures comprise multi-panel layouts. We adopt the constituent-panel segmentation method described by Li et al. [22]. Rather than relying on gap-based heuristics, which are prone to under- or over-segmentation, this method identifies subgraph boundaries by grouping pixels according to intensity distributions and computing the minimal bounding box around each panel. Prior evaluations have shown that it outperforms gap-based segmentation in accuracy and robustness [22]. Each segmented subfigure is then classified by a CNN to determine whether it contains a bar chart. Although several transfer-learning architectures are available for image classification, including VGG, ResNet, DenseNet, Xception, and EfficientNet variants, we selected Google’s Inception v3 architecture for three reasons [23]. First, the goal of this stage was panel-level figure-type classification rather than fine-grained object detection, so an image-classification backbone was appropriate and more computationally efficient than object-detection architectures such as Faster R-CNN or YOLO [24]. Second, Inception v3 uses multi-scale convolutional modules and factorized convolutions, allowing it to capture visual features at different spatial scales while remaining computationally efficient—well suited to scientific figures, in which discriminative features such as bars, axes, legends, tick marks, and labels vary substantially in size, density, and layout across publications. Third, because our curated corpus of scientific visualizations was modest in size, transfer learning from an ImageNet-pretrained model reduced the need to train a deep CNN from scratch while still allowing the final layers to be adapted to domain-specific figure categories [25]. We therefore repurposed the ImageNet-pretrained Inception v3 model for figure-type classification by replacing and retraining its final classification layers on a curated corpus of bar charts, line graphs, scatter plots, and non-chart panels. The retrained classifier was applied to all segmented panels, and only those identified as bar charts advanced to downstream processing. Panels containing horizontally oriented bar charts were rotated 90° counterclockwise to normalize orientation prior to image disassembly. Because the objective of this study was to evaluate the feasibility of end-to-end bar-chart data extraction rather than to benchmark CNN backbones, we did not perform a systematic architecture comparison; this is noted as a limitation and a direction for future work.

### Text detection

The second step uses OCR to extract textual elements from each bar chart, including axis labels, tick values, and numeric annotations. Prior to OCR, each image undergoes preprocessing designed to enhance text visibility and suppress non-text regions. We first apply Otsu’s global threshold method to binarize the image and improve contrast between foreground text and background. We then use a border-following contour-detection algorithm to identify and remove large graphical elements—such as the bars themselves and thin gridlines—whose presence can substantially degrade OCR performance [26]. The remaining contours correspond to candidate text regions, from which we construct a binary text mask that preserves only the bounding boxes likely to contain textual content.

To further improve OCR accuracy, the text mask is up-scaled by a factor of two using Waifu2x, a deep CNN–based super-resolution and de-noising algorithm [27]. Prior studies have shown that Waifu2x achieves superior reconstruction quality and downstream OCR performance compared with widely used super-resolution techniques, including the Zeyde and A+ dictionary-learning–based methods [28–30]. After up-scaling, text is recognized using Tesseract OCR, an open-source engine that supports multilingual character sets and has been widely applied in biomedical informatics and document-analysis workflows [31].

### Image disassembly

The third stage isolates the structural components of each bar chart (e.g., axes, bar regions, and gridlines) to enable accurate extraction of quantitative information. We first remove all textual elements from the image using the text mask generated during OCR preprocessing, producing a text-free chart suitable for structural analysis.

To identify the chart axes, we apply Canny edge detection followed by the probabilistic Hough transform, which detects dominant line segments within the image [32,33]. Among the detected lines, the two longest segments that intersect at approximately 90° are selected as the x- and y-axes. The image is then cropped to the rectangular region defined by these axes to remove extraneous whitespace and figure decorations.

Gridlines are removed using a morphological opening operation with a kernel size of 5, a parameter chosen empirically based on performance across a curated corpus of training figures extracted from published manuscripts [34]. After gridline removal, we identify contour corners using a chain-approximation algorithm that simplifies contour boundaries while preserving geometric structure. Vertical line candidates are formed by pairing corners with identical x-coordinates. Bars are then reconstructed by grouping pairs of adjacent vertical lines that share contour vertices with the same y-coordinate. The pairing procedure begins at a corner lying on the x-axis and terminates when the matched contour path returns to the x-axis, ensuring complete enclosure of each bar region.

For quantitative extraction, a small central slice of each identified bar is sampled and used for template and color matching. Bars with similar color profiles and template characteristics are grouped, facilitating multi-series interpretation and enabling accurate mapping between legend entries and bar clusters.

### Data extraction

The final stage reconstructs numerical data by integrating recognized text with the structural components extracted from the preprocessed image, generating a complete tabular representation of the chart. Textual elements are first classified according to their orientation and spatial relationship to chart features. Text aligned perpendicular to the y-axis is assigned as y-tick values, whereas text parallel to the x-axis is assigned as x-tick values. Axis labels are identified by locating text positioned immediately to the left (or right, where chart orientation required rotation) of the y-tick values, and text located below the x-tick values.

Bar values are extracted using a two-tiered approach. When a bar carries a directly annotated numeric label, typically placed above the bar, this value is parsed and used as the definitive measurement. Because academic bar charts often omit per-bar annotations, the pipeline also includes a numeric approximation method. In these cases, we estimate bar values from a pixel-to-numeric mapping derived from the average pixel distance between consecutive y-tick labels. This linear interpolation infers the quantitative value represented by each bar from its measured height or length.

**Fig 2:**
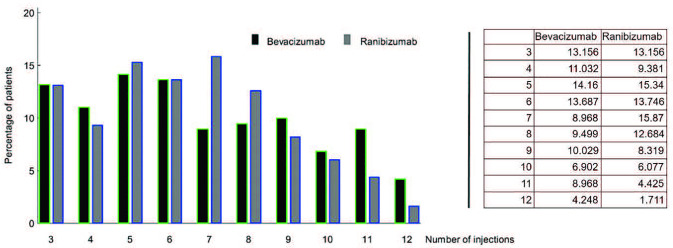
Data extraction using the automated pipeline.

This procedure assumes a linear y-axis scale and is not applicable to charts plotted on logarithmic or other nonlinear scales. Fig 2 illustrates an automatically generated data table in which per-bar values were estimated using this interpolation-based method.

### Validation

**Fig 3:**
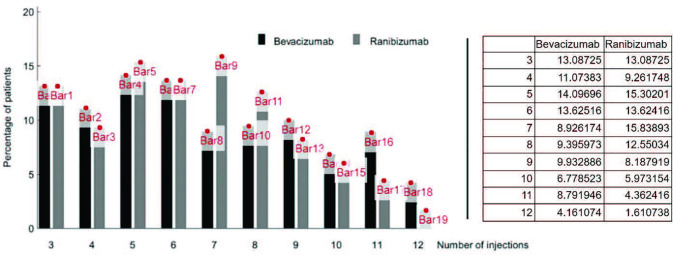
Data extraction obtained semi-manually using WebPlotDigitizer.

To evaluate the proposed pipeline, we randomly selected ten PubMed Central articles published within the previous ten years that reported randomized controlled trials (RCTs) of age-related macular degeneration (AMD) [35–44]. Across these articles, we identified 58 figures, of which 28 were bar charts. All bar charts were independently annotated by two reviewers (IL and RTY) using WebPlotDigitizer (version 4.2; Ankit Rohatgi, Austin, Texas, United States of America, 2019), a semi-automated tool widely used for extracting quantitative data from static scientific figures [45]. Reviewers manually specified two reference y-axis values and marked the top of each bar to estimate its corresponding numeric value, as illustrated in Fig 3. Discrepancies between the two sets of measurements were resolved by averaging the independently obtained values, which served as the ground-truth benchmark. We used WebPlotDigitizer to establish the manual reference standard because it is widely adopted for static figures; more recent annotation platforms such as the Computer Vision Annotation Tool (CVAT) may be better suited to large-scale, automated annotation of heterogeneous chart types and are a useful option for future scaling [46]. The same set of bar charts was then processed through the automated pipeline for comparative evaluation.

### Data analysis

To evaluate concordance between the manually derived benchmark values and the automated extractions, we conducted a Bland–Altman analysis. This method assesses agreement by quantifying the mean difference (bias) between two measurement techniques and the limits of agreement, defined as the bias ± 1.96 standard deviations [47]. Under the assumption of approximate normality, 95% of the differences between the two methods are expected to fall within this interval. Agreement proportions are reported with 95% confidence intervals (CIs) computed using the Wilson score method, and CIs for the Bland–Altman bias and limits of agreement were derived using the standard normal-approximation formulas [47]. For the illustrative meta-analysis, we examined changes in visual acuity (VA), operationalized as the proportion of patients who lost fewer than 15 letters on the Early Treatment Diabetic Retinopathy Study (ETDRS) chart following one of three regimens: bevacizumab 1.25 mg monthly, ranibizumab 0.5 mg monthly, or ranibizumab 0.5 mg under a treat-and-extend protocol [48].

### Ethics approval and consent to participate

This study analyzed only previously published figures and used no human-subjects data.

## Results

Of the 28 bar charts scrubbed from the PDF articles, 26 (92.9%; 95% CI, 77.4–98.0) were correctly recognized by the CNN-based Inception v3 classifier. Among these 26 charts, 96.0% of text blocks (599 of 624; 95% CI, 94.2–97.3) and 79.1% of bars (292 of 369; 95% CI, 74.7–83.0) were correctly detected. Qualitative examination showed that the morphological opening operation tended to fail to identify bars on charts with dense gridlines.

Table 1 reports the agreement between manually and automatically extracted results. An exact match for labels and tick values means that the automatically extracted string was identical to the manually extracted one. For bar values, the agreement represents the percentage of values within ±5%, ± 2%, and ±1% of the manually extracted value, reported with 95% CIs.

**Table 1:** Agreement between manually and automatically extracted data.

Measure	Result	95% CI
Figure-type recognition (Inception v3)	26/28 (92.9%)	77.4–98.0
Text-block detection	599/624 (96.0%)	94.2–97.3
Bar detection	292/369 (79.1%)	74.7–83.0
Bar values within ±5%	237/292 (81.2%)	76.3–85.2
Bar values within ±2%	184/292 (63.0%)	57.3–68.3
Bar values within ±1%	142/292 (48.6%)	43.0–54.3

**Fig 4:**
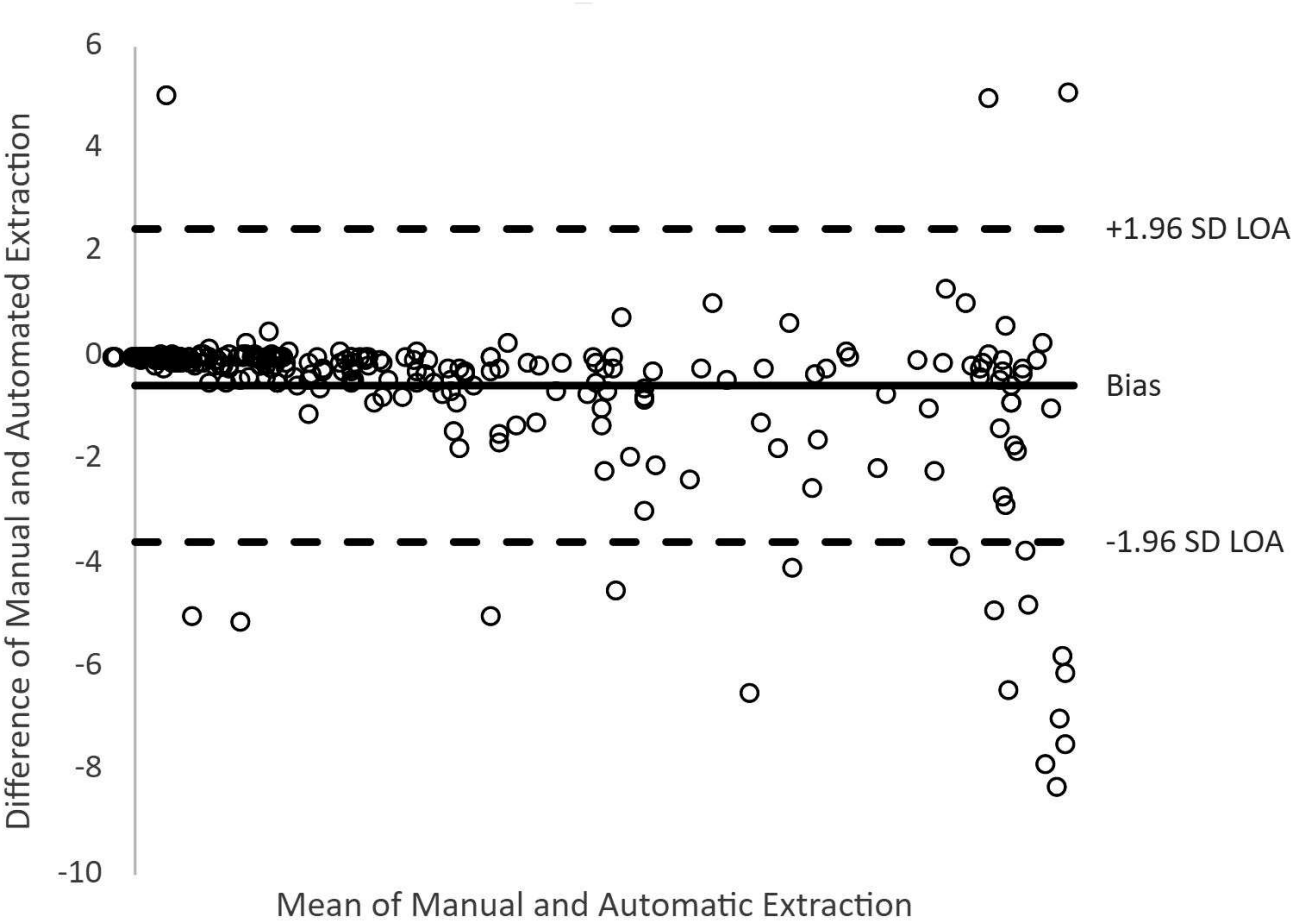
Results of the Bland–Altman analysis.

**Fig 5:**
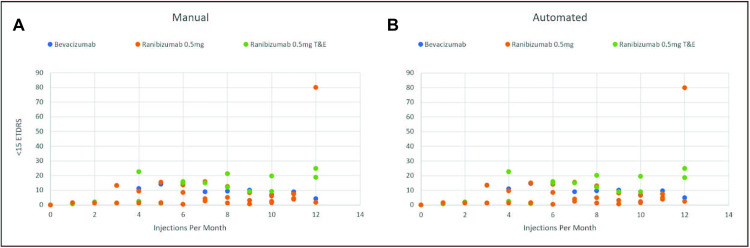
Changes in ETDRS letters detected in the sample meta-analysis (manual vs. automated).

Bland–Altman analysis was performed on the 292 successfully extracted data points, excluding the 77 points that were not detected during extraction (Fig 4). The mean difference (bias) between manual and automated values was −0.18 (95% CI, −0.34 to −0.02), with a standard deviation of 1.41, giving 95% limits of agreement of −2.94 (95% CI, −3.22 to −2.67) to 2.58 (95% CI, 2.31 to 2.86). Overall, 94.9% of differences (277 of 292) fell within these limits. A side-by-side comparison of manually and automatically extracted data is shown in Fig 5; these scatterplots show the percentage of patients in each treatment arm who lost fewer than 15 letters on the ETDRS chart over the 12-month post-treatment period.

## Discussion

Manual extraction of quantitative data from scientific figures remains a time-consuming and error-prone barrier to evidence synthesis, particularly as the pace of biomedical publication continues to accelerate. Although several computational approaches have attempted to automate figure interpretation, existing systems have not yet demonstrated sufficient accuracy or robustness for routine use in meta-analysis workflows. In this study, we present a multi-stage pipeline that integrates CNN-based figure classification, OCR, image processing, and heuristic reasoning to extract numerical values from bar charts embedded in published articles. By targeting figure-embedded data, the pipeline complements existing approaches focused primarily on text and tables and represents a step toward more complete automation of biomedical evidence extraction.

The practical motivation for this work is not only technical chart recognition but also the downstream burden of evidence synthesis. In systematic reviews, missing or hard-to-extract numerical values can slow the review, introduce human error, or force the exclusion of otherwise relevant studies. Automating extraction from bar-chart images could help reviewers recover data that are not reported in tables or text, improve reproducibility by standardizing the extraction procedure, and reduce the manual effort required to prepare data for meta-analysis. Although the current pipeline is limited to bar charts and linear axes, it demonstrates how computer-vision methods can begin to bridge the gap between image-based scientific reporting and structured evidence synthesis.

Our empirical validation focused on randomized controlled trials of age-related macular degeneration (AMD), a disease area of substantial clinical and research importance. Across the evaluation set, the pipeline demonstrated strong performance, including 92.9% figure-type recognition accuracy, 96.0% text-block detection accuracy, and 79.1% bar detection accuracy. This represents a considerable improvement over the 39.81% bar-panel recognition rate reported by He et al., underscoring the benefit of our constituent-panel segmentation and preprocessing strategy [9]. By contrast, the proportion of extracted values within ±1% error (48.6%) was lower than the 64.75% image-recognition rate reported by Liu et al., whose relational-network model learns coordinate–value relationships directly from large numbers of charts [7]. These contrasts underscore the limitations of static, rule-based heuristics on visually heterogeneous chart designs and motivate the integration of learning-based relational reasoning. Table 2 situates our results among representative prior systems; because these systems were evaluated on different datasets, chart populations, and metrics, the comparison provides descriptive context rather than a controlled head-to-head benchmark.

**Table 2:** Comparison with representative chart data-extraction systems.

System (year)	Domain	Approach	Reported performance
Huang & Tan (2007)	General infographics	Rule-based + classification	93.75% graphics, 88.22% text-block (bar charts)
He et al. (2018)	Biomedical (PMC)	Rules + CNN	39.81% bar-panel recognition
Liu et al. (2019)	General charts	Faster R-CNN + relational net	64.75% image recognition; coordinate–value mapping
ChartOCR (2021)	General (ExcelChart400K)	Deep keypoints + rules	Data extraction across bar/line/pie
BarChartAnalyzer (2022)	General bar charts	Tensor voting + CNN (semi-automated)	Data table extraction + summarization
Cardaras et al. (2026) [This Study]	Biomedical (AMD RCTs, real figures)	Heuristics + Inception-v3 (end-to-end to table)	92.9% figure / 96.0% text / 79.1% bar; 81/63/49% within ±5/2/1%

The Bland–Altman analysis indicated a small negative bias in automatically extracted values relative to manually extracted values and identified a limited number of outliers. Most outliers originated from OCR misclassification, particularly confusion between “2” and “7”, highlighting the sensitivity of downstream numerical reconstruction to OCR fidelity. To address this, we implemented a lightweight numeric post-correction step that flags values falling outside the chart’s axis range and re-scores them against digit-confusion candidates. On the 20-bar example shown in Figs 2 and 3, this step preserved all already-plausible values without introducing false corrections while rejecting physically impossible readings; a fuller evaluation on larger corpora, together with domain-specific OCR fine-tuning and language-model–based numeric correction, is a promising route to further reducing these errors [49].

Our work builds on prior systems for interpreting scientific figures. Huang and Tan demonstrated strong graphics and text recognition using a modular, classification-centered architecture [8], while He et al. combined rule-based processing with a CNN for bar-segment detection but achieved limited panel-level accuracy [9]. Liu et al. advanced a relational-network approach that learns coordinate–value mappings directly from thousands of diverse figures [7], and more recent deep-learning systems—including ChartOCR, ChartQA, and DePlot—frame chart understanding as an end-to-end learning or plot-to-table translation problem [10–12]. Semi-automated tools such as BarChartAnalyzer integrate chart-type classification, object and text detection, and tensor-voting–based extraction, and extend to chart summarization and redesign [13,14]. Relative to these systems, the contribution of the present work is not a new recognition architecture but an end-to-end, predominantly interpretable pipeline validated on real, heterogeneous biomedical figures and quantitatively benchmarked against a manual reference standard using Bland–Altman agreement, an evaluation oriented toward the precision requirements of evidence synthesis rather than toward chart question answering or summarization. Collectively, these studies and our results show both the promise and the constraints of automated chart interpretation: achieving fully generalizable performance across the diverse bar-chart designs found in the biomedical literature will most likely require architectures that combine structured heuristics with deep learning–based relational reasoning.

A practical advantage of the present design is interpretability. Because data reconstruction is performed by explicit geometric and textual rules rather than a single opaque model, every extracted value is traceable to concrete intermediate evidence including the detected axis lines, the calibrated pixel-to-value mapping, the reconstructed bar boundaries, and the specific OCR tokens used. Errors can therefore be localized to a particular stage and inspected, which is valuable for evidence synthesis, where the provenance and auditability of each extracted datum directly affect trust in downstream meta-analytic estimates. The only learned component, the Inception v3 figure-type classifier, acts as a transparent gate at the panel level: its decisions can be reviewed per panel, and its misclassifications are directly observable in the recognition statistics. This contrasts with fully end-to-end deep models, which can offer higher accuracy but provide limited insight into how individual numbers are produced. Combining the transparency of these heuristics with the robustness of learning-based components, while preserving traceability, is an important goal for future systems, and uncertainty-aware or explainable classification methods may help flag low-confidence extractions for human review [49,50].

This study has several limitations. First, validation was restricted to a single disease domain (AMD) and a relatively small set of publications (28 bar charts from ten articles), which limits generalizability; large-scale, multi-domain evaluation will be necessary to establish robustness across the visual conventions of different fields, and such a multi-domain annotated corpus was not available for this proof-of-concept. Second, the current pipeline processes only bar charts, excluding other chart types such as line graphs, scatter plots, Kaplan–Meier curves, and forest plots; supporting these will require additional feature extractors and relationship models. Third, bar localization relies on classical morphological operations, which degraded on charts with dense gridlines; instance-segmentation models such as Mask R-CNN are a promising alternative for more robust bar localization, although training and validating such a model would require a substantial annotated bar-chart corpus beyond the scope of this study [51]. Fourth, although Inception v3 was selected as a computationally efficient and well-supported transfer-learning backbone, we did not systematically compare it against alternatives such as ResNet, DenseNet, Xception, EfficientNet, or transformer-based vision models; future work should benchmark multiple backbones and evaluate their robustness to image noise and augmentation on larger, multi-domain datasets [49,50]. Finally, the pipeline assumes linear y-axis scaling and cannot yet process logarithmic or other nonlinear axes, which are common in laboratory and preclinical studies.

Despite these limitations, this work demonstrates the feasibility of automating data extraction directly from published figures using real-world, heterogeneous images rather than simulated graphics. Although universal adoption of machine-readable data formats or mandatory deposition of raw data alongside publications would be preferable, automated figure analysis offers an important interim solution that reduces human workload and extraction errors in meta-analytic research. Future work should focus on expanding chart-type coverage, integrating neural relational reasoning, improving OCR robustness through domain-specific fine-tuning and language-model–based correction, and validating performance on larger, multi-domain corpora, with the goal of building a high-accuracy, fully automated system for quantitative evidence synthesis across the biomedical literature.

## Conclusions

In this study, we developed and validated a computational pipeline for automated extraction of quantitative data from bar charts, one of the most prevalent figure types in biomedical publications. By integrating hand-engineered heuristics with CNN-based classification, the approach enables reliable identification of chart components and accurate reconstruction of underlying numerical values from rasterized figures embedded in PDF documents. Evaluation on manually annotated PubMed Central articles on age-related macular degeneration demonstrated strong performance across multiple feature-recognition and data-reconstruction tasks, supporting the technical validity of the pipeline.

Beyond this specific disease context, the proposed method addresses a broader challenge in biomedical evidence synthesis: important quantitative results are frequently reported only in graphical form, making them difficult to extract at scale. By converting bar-chart images into structured numerical data, the pipeline can reduce manual extraction burden, improve reproducibility, and enhance the scalability of systematic reviews and meta-analyses as the published literature continues to grow. Although the current implementation is limited to bar charts and linear axes, the underlying architecture is disease-agnostic and extensible to other chart types and clinical domains. Future work should focus on expanding figure coverage, improving robustness across diverse visual designs, and validating performance on larger, multi-domain corpora, with the goal of enabling high-throughput, automated data extraction that supports timely and rigorous evidence-based decision-making.

## Supporting information

S1 File Data and code v2. (ZIP)
