# Supplementary figures and images for: A computer vision-based approach for automatically extracting data from bar chart raster images to facilitate meta-analysis of biomedical literature
Source: PLoS One. 2026 Jul 31;21(7):e0347081. doi: 10.1371/journal.pone.0347081 (PMC13426920; doi:10.1371/journal.pone.0347081)

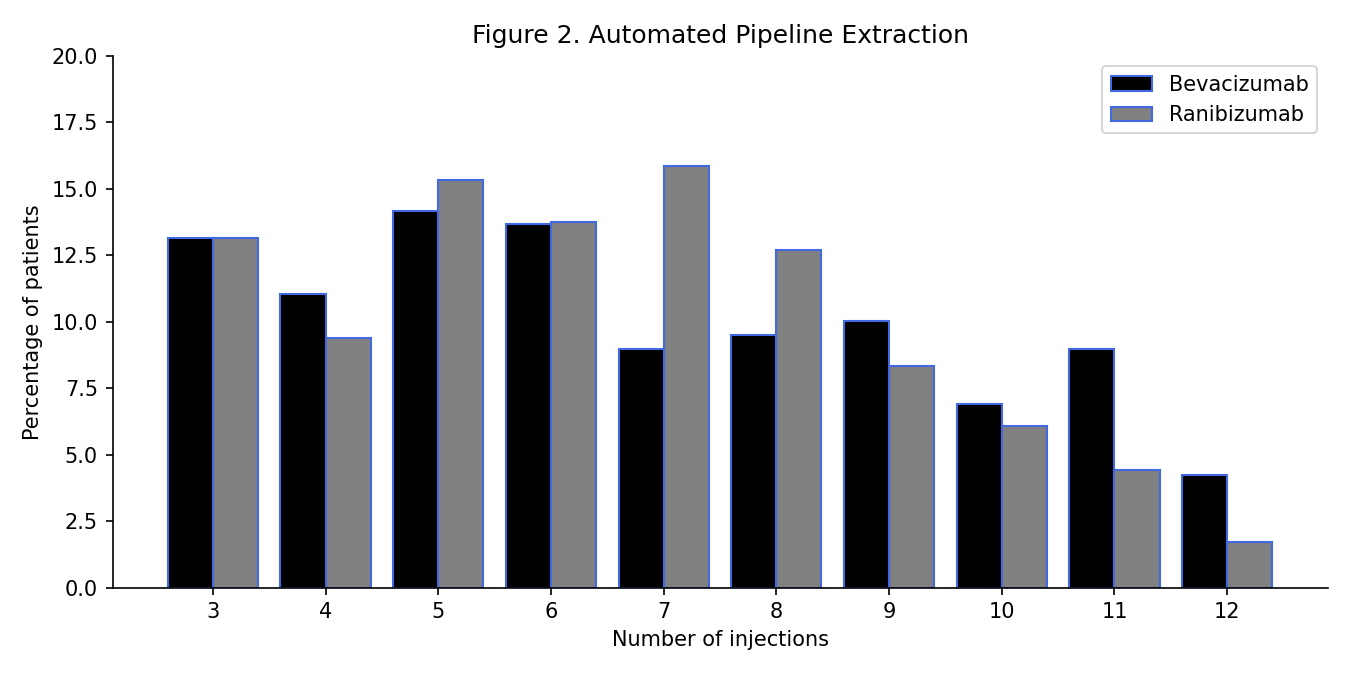

Supplement: S1 File — (ZIP) [file pone.0347081.s001.zip › Data and Code_v2/figures_out/figure2_automated.png]

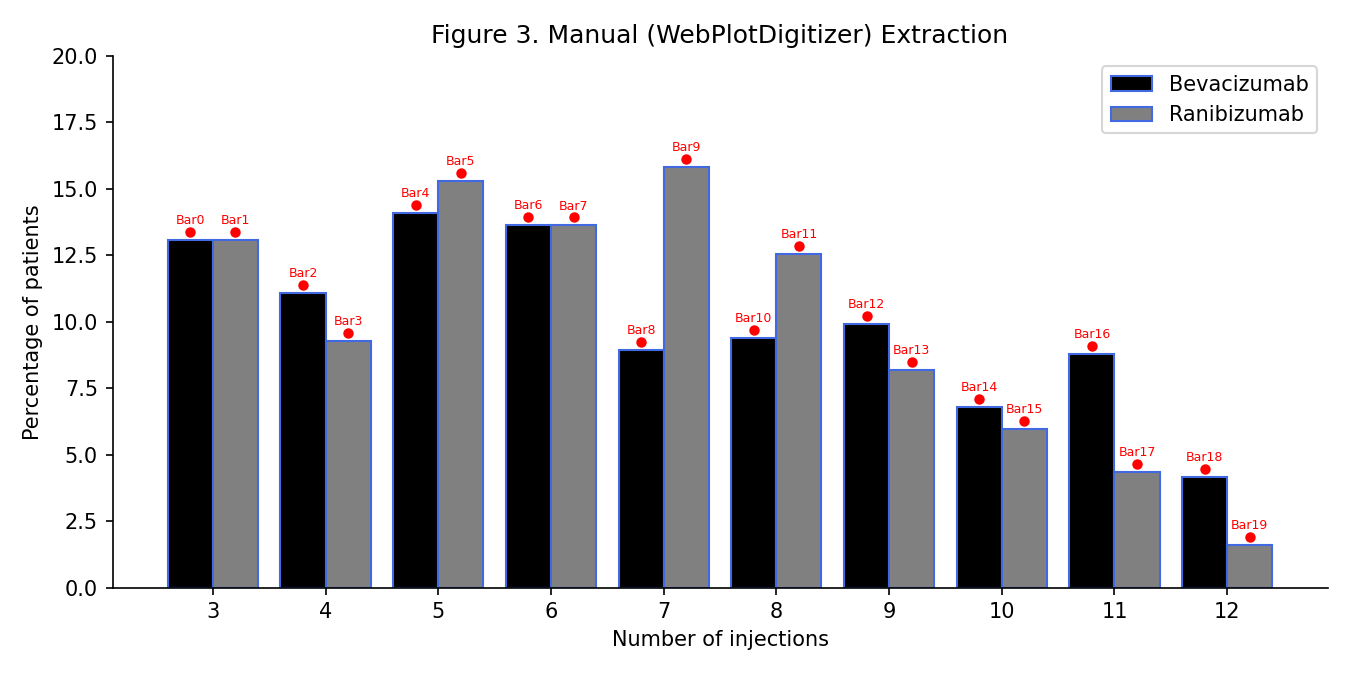

Supplement: S1 File — (ZIP) [file pone.0347081.s001.zip › Data and Code_v2/figures_out/figure3_manual.png]

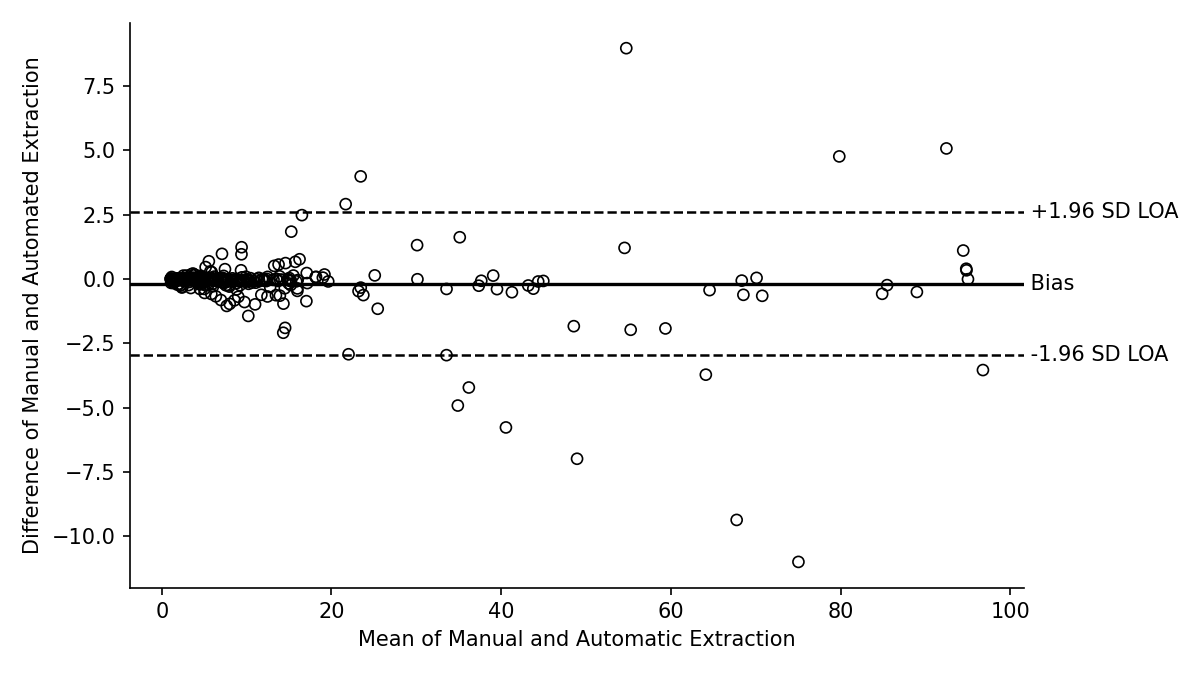

Supplement: S1 File — (ZIP) [file pone.0347081.s001.zip › Data and Code_v2/figures_out/figure4_bland_altman.png]

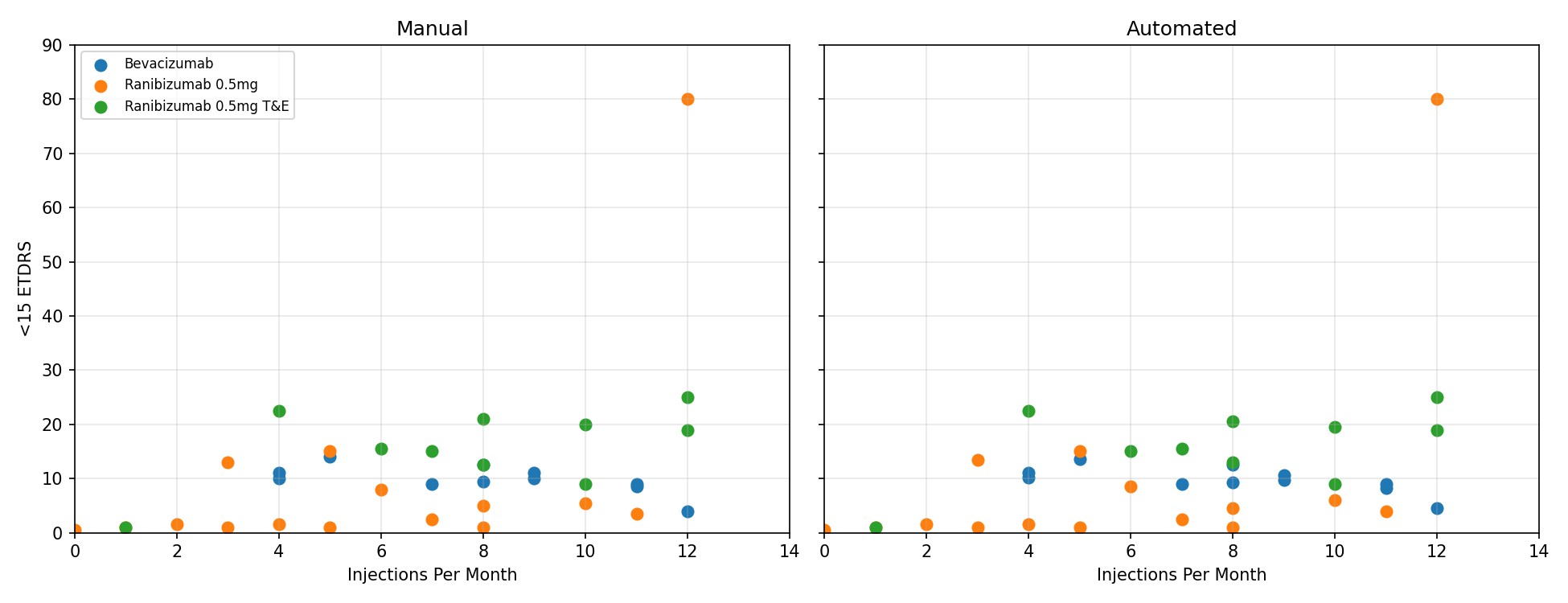

Supplement: S1 File — (ZIP) [file pone.0347081.s001.zip › Data and Code_v2/figures_out/figure5_meta_analysis.png]
